# Inter hospital external validation of interpretable machine learning based triage score for the emergency department using common data model

**DOI:** 10.1038/s41598-024-54364-7

**Published:** 2024-03-20

**Authors:** Jae Yong Yu, Doyeop Kim, Sunyoung Yoon, Taerim Kim, SeJin Heo, Hansol Chang, Gab Soo Han, Kyung Won Jeong, Rae Woong Park, Jun Myung Gwon, Feng Xie, Marcus Eng Hock Ong, Yih Yng Ng, Hyung Joon Joo, Won Chul Cha

**Affiliations:** 1 Department of Biomedical Systems Informatics, Yonsei University College of Medicine, Seoul, Republic of Korea; 2 Department of Biomedical Informatics, Ajou University School of Medicine, Suwon, Republic of Korea; 3 Department of Digital Health, Samsung Advanced Institute for Health Science & Technology, Sungkyunkwan University, Seoul, Republic of Korea; 4 Department of Emergency Medicine, Samsung Medical Center, Sungkyunkwan University School of Medicine, 115 Irwon-ro Gangnam-gu, Seoul, 06355 Republic of Korea; 5 Department of Cardiology, Cardiovascular Center, College of Medicine, Korea University, Seoul, Republic of Korea; 6 Department of Critical Care and Emergency Medicine, Mediplex Sejong Hospital, Incheon, Republic of Korea; 7 Programme in Health Services and Systems Research, Duke–National University of Singapore Medical School, Singapore, Singapore; 8 Department of Biomedical Data Science, Stanford University, Stanford, USA; 9 Department of Anesthesiology, Perioperative, and Pain Medicine, Stanford University, Stanford, USA; 10 Department of Emergency Medicine, Singapore General Hospital, Singapore, Singapore; 11 Digital and Smart Health Office, Tan Tock Seng Hospital, Singapore, Singapore; 12 Digital Innovation Center, Samsung Medical Center, Seoul, Republic of Korea; 13 Department of Biomedical Sciences, Ajou University Graduate School of Medicine, Suwon, Republic of Korea

**Keywords:** Computer science, Outcomes research

## Abstract

Emergency departments (ED) are complex, triage is a main task in the ED to prioritize patient with limited medical resources who need them most. Machine learning (ML) based ED triage tool, Score for Emergency Risk Prediction (SERP), was previously developed using an interpretable ML framework with single center. We aimed to develop SERP with 3 Korean multicenter cohorts based on common data model (CDM) without data sharing and compare performance with inter-hospital validation design. This retrospective cohort study included all adult emergency visit patients of 3 hospitals in Korea from 2016 to 2017. We adopted CDM for the standardized multicenter research. The outcome of interest was 2-day mortality after the patients’ ED visit. We developed each hospital SERP using interpretable ML framework and validated inter-hospital wisely. We accessed the performance of each hospital’s score based on some metrics considering data imbalance strategy. The study population for each hospital included 87,670, 83,363 and 54,423 ED visits from 2016 to 2017. The 2-day mortality rate were 0.51%, 0.56% and 0.65%. Validation results showed accurate for inter hospital validation which has at least AUROC of 0.899 (0.858–0.940). We developed multicenter based Interpretable ML model using CDM for 2-day mortality prediction and executed Inter-hospital external validation which showed enough high accuracy.

## Introduction

Emergency department (ED) is complex and need urgent judgement for the better triage^1,2^. In order to determine the patient’s condition quickly, Korea Triage Acuity Scale (KTAS), New Early Warning Score and Modified Early Warning Score have been developed by expertise^3,4^. However, although most scores require complicated process to make, they are fixed score and have low reliability and poor outcome due to subjective assessment^5^. To solve this problem, data and machine learning (ML) based objective score has emerged^6,7^.

Those ML based models have problems of black box and external validation^8,9^. There has been some studies for interpretable triage in ED which utilized framework for interpretable scoring system called Autoscore^10–12^. However it was only conducted with limited population and specific for ER admission patients^11^. Each hospital have different population and characteristics, so we need to develop each hospital based unique score for the application.

Another tricky part for the external validation in ML research is data protection law and policy^13,14^. It is impossible to transfer the data into other hospital for preserving privacy. To solve this challenge, common data model (CDM) can be adopted for each hospital^15^. Through the CDM format, multicenter research could be done without data transfer. Standardized format of terminology and structure can be made for each hospital’s different electronic medical records format and policy. There has been some CDM based research regarding the ML^16,17^, there was no CDM based interpretable machine learning research in Korea.

The aim of the study is to develop, and inter-hospital external validate the interpretable ML score among the 3 big hospitals in Korea using novel framework using CDM.

## Results

**Figure 1:**
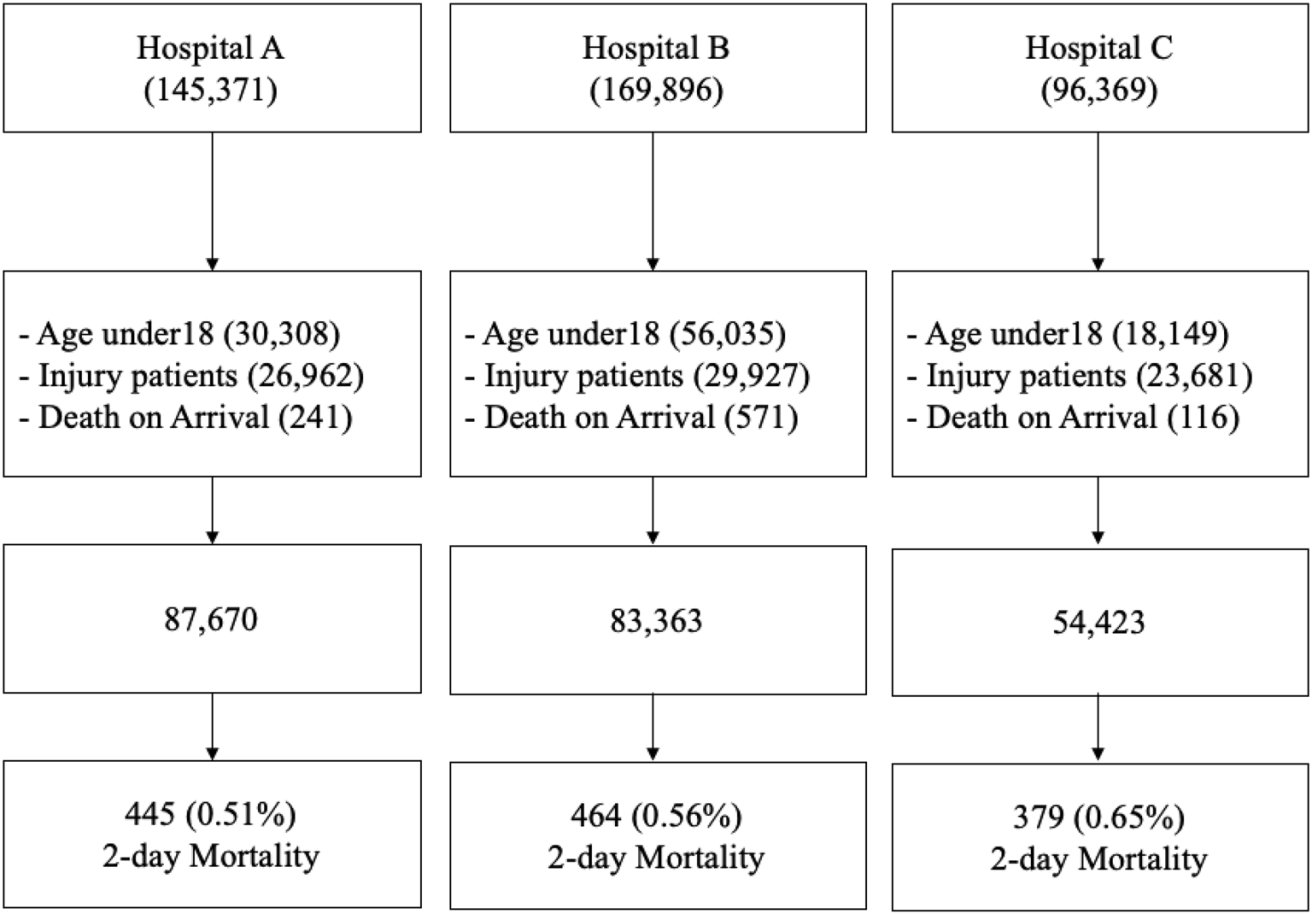
Flow chart for each hospital from 2016 to 2017 emergency department visits. Age under 18, traumatic and death on arrival patient were excluded.

During the same study period for each hospital from 2016 to 2017 145,371, 169,896 and 96,369 patients visited ED in A, B and C respectively as shown in Fig. 1. Among them, totally 57,511, 86,533 and 41,946 patients were excluded due to age under 18, DOA, and trauma patient. Finally, 86,670, 83,363 and 54,423 patients were used for developing models. The mortality rate was from 0.51%, 0.55% and 0.65% for 2 days.

The distribution of ED patients’ demographics for each hospital is shown in Table 1. Each cohort included 445, 464 and 379 of events. (67.2 (14.3), 72.8 (14.4) and 72.5 (13.5) for age; 265 (59.6.%), 245 (52.8%) and 218 (57.5%) for male). Regarding the mortality patient, there were quite differences between hospitals, especially in patient conciseness of Alert at hospital A (70.8%) have higher than others (44.0 and 28.2%). Moreover, patient with severe (KTAS1 or KTAS2) at scene in hospital C (87.4%) was higher than other hospitals. (49.7% and 71.3%). Regarding the vital sign all hospital have different patterns, especially in SPO2 and BP. In terms of comorbidities history, Hospital A have much higher cancer related patients (73.9%) compared to B and C (9.5 and 5%). Whereas Hospital B and C have higher chronic disease including diabetes (28.2 and 28%). Synthetic minority over-sampling technique (SMOTE) based distribution and significance of difference for each variable were provided with standardized mean difference (SMD) were shown in Supplementary Tables 1–3.

**Table 1:** Baseline Demographic for each hospital ED triage information from 2016 to 2017.

	Hospital A			Hospital B			Hospital C		
	No death (n = 87,225)	2 d- mortality (n = 445)	*p*-value	No death (n = 82,899)	2 d- mortality (n = 464)	*p*-value	No death (n = 54,044)	2 d- mortality (n = 379)	*p*-value
Sex			< 0.001			0.019			< 0.001
Male	42,608 (48.8%)	265 (59.6%)		39,155 (47.2%)	245 (52.8%)		24,276 (44.9%)	218 (57.5%)	
Female	44,617 (51.2%)	180 (40.4%)		43,744 (52.8%)	219 (47.2%)		29,768 (55.1%)	161 (42.5%)	
Age, mean (SD)	55.3 ± 17.5	67.2 ± 14.3	< 0.001	51.4 ± 19.1	72.8 ± 14.4	< 0.001	51.7 ± 20.4	72.5 ± 13.6	< 0.001
Day of week			0.202			0.909			0.288
Midweek	36,174 (41.5%)	187 (42.0%)		33,341 (40.2%)	186 (40.1%)		21,498 (39.8%)	156 (41.2%)	
Weekend	24,699 (28.3%)	137 (30.8%)		25,376 (30.6%)	143 (30.8%)		17,053 (31.6%)	105 (27.7%)	
Friday	12,147 (13.9%)	47 (10.6%)		11,331 (13.7%)	59 (12.7%)		7334 (13.6%)	48 (12.7%)	
Monday	14,205 (16.3%)	74 (16.6%)		12,851 (15.5%)	76 (16.4%)		8159 (15.1%)	70 (18.5%)	
Shift time			0.002			0.129			0.217
8 am to 4 pm	40,217 (46.1%)	218 (49.0%)		35,176 (42.4%)	200 (43.1%)		20,596 (38.1%)	163 (43.0%)	
4 pm to midnight	31,533 (36.2%)	128 (28.8%)		30,223 (36.5%)	183 (39.4%)		20,974 (38.8%)	128 (33.8%)	
Midnight to 8 am	15,475 (17.7%)	99 (22.2%)		17,500 (21.1%)	81 (17.5%)		12,474 (23.1%)	88 (23.2%)	
Triage categories			< 0.001			< 0.001			< 0.001
1 (most severe)	564 (0.6%)	73 (16.4%)		506 (0.6%)	84 (18.1%)		665 (1.2%)	197 (52.0%)	
2	7904 (9.1%)	148 (33.3%)		8809 (10.6%)	247 (53.2%)		7362 (13.6%)	134 (35.4%)	
3	40,658 (46.6%)	177 (39.8%)		56,088 (67.7%)	127 (27.4%)		34,072 (63.0%)	46 (12.1%)	
4	31,387 (36.0%)	45 (10.1%)		13,127 (15.8%)	4 (0.9%)		8986 (16.6%)	2 (0.5%)	
5 (less severe)	6712 (7.7%)	2 (0.4%)		4369 (5.3%)	2 (0.4%)		2959 (5.5%)	0 (0.0%)	
Consciousness			< 0.001			< 0.001			< 0.001
Alert	84,969 (97.4%)	315 (70.8%)		79,268 (95.6%)	204 (44.0%)		50,943 (94.3%)	107 (28.2%)	
Verbal	1345 (1.5%)	50 (11.2%)		1980 (2.4%)	88 (19.0%)		2277 (4.2%)	68 (17.9%)	
Painful	773 (0.9%)	57 (12.8%)		1485 (1.8%)	124 (26.7%)		723 (1.3%)	66 (17.4%)	
Unconsciousness	138 (0.2%)	23 (5.2%)		166 (0.2%)	48 (10.3%)		101 (0.2%)	138 (36.4%)	
Route of arrival			< 0.001			< 0.001			< 0.001
Direct	69,632 (79.8%)	282 (63.4%)		65,011 (78.4%)	216 (46.6%)		46,039 (85.2%)	289 (76.3%)	
Other*	17,593 (20.2%)	163 (36.6%)		17,888 (21.6%)	248 (53.4%)		8005 (14.8%)	90 (23.7%)	
Mode of transport			< 0.001			< 0.001			< 0.001
Ambulance	17,678 (20.3%)	317 (71.2%)		17,454 (21.1%)	350 (75.4%)		16,353 (30.3%)	331 (87.3%)	
Other*	69,547 (79.7%)	128 (28.8%)		65,445 (78.9%)	114 (24.6%)		37,691 (69.7%)	48 (12.7%)	
Vital signs, mean (SD)									
Pulse, /min	89.5 ± 20.1	108.0 ± 25.4	< 0.001	87.9 ± 17.6	102.2 ± 28.1	< 0.001	89.3 ± 19.0	100.8 ± 22.0	< 0.001
Blood pressure, mm Hg									
Systolic	129.9 ± 25.0	118.0 ± 32.3	< 0.001	131.7 ± 24.4	105.7 ± 30.5	< 0.001	134.1 ± 24.4	112.6 ± 31.8	< 0.001
Diastolic	77.1 ± 15.4	68.1 ± 20.1	< 0.001	78.8 ± 15.4	63.0 ± 20.0	< 0.001	81.9 ± 15.3	73.3 ± 21.2	< 0.001
Respiration, /min	19.0 ± 2.4	22.7 ± 5.4	< 0.001	16.2 ± 3.1	22.0 ± 6.3	< 0.001	20.9 ± 2.9	22.0 ± 7.2	0.034
SPo2, %	97.3 ± 3.2	91.8 ± 9.8	< 0.001	98.4 ± 2.2	93.3 ± 8.0	< 0.001	97.5 ± 3.0	88.9 ± 12.2	< 0.001
Temperature, °C	37.0 ± 0.8	36.8 ± 1.0	< 0.001	36.8 ± 0.7	36.6 ± 1.2	0.008	36.8 ± 0.8	36.1 ± 1.1	< 0.001
Comorbidity									
Myocardial infarction	1336 (1.5%)	16 (3.6%)	0.001	1240 (1.5%)	22 (4.7%)	< 0.001	1075 (2%)	23 (6.1%)	< 0.001
Congestive heart failure	4324 (5%)	38 (8.5%)	0.001	2042 (2.5%)	47 (10.1%)	< 0.001	1349 (2.5%)	21 (5.5%)	< 0.001
Peripheral vascular disease	2065 (2.4%)	14 (3.1%)	0.357	682 (0.8%)	11 (2.4%)	0.001	508 (0.9%)	5 (1.3%)	0.621
Stroke	7641 (8.8%)	48 (10.8%)	0.155	4835 (5.8%)	48 (10.3%)	< 0.001	2723 (5%)	24 (6.3%)	0.304
Dementia	2860 (3.3%)	26 (5.8%)	0.004	905 (1.1%)	15 (3.2%)	< 0.001	1235 (2.3%)	25 (6.6%)	< 0.001
Chronic pulmonary disease	6121 (7%)	55 (12.4%)	< 0.001	3820 (4.6%)	39 (8.4%)	< 0.001	1800 (3.3%)	20 (5.3%)	0.050
Rheumatoid disease	1152 (1.3%)	5 (1.1%)	0.877	800 (1%)	5 (1.1%)	0.993	390 (0.7%)	3 (0.8%)	1.000
Diabetes without complications	3427 (3.9%)	18 (4%)	0.997	2281 (2.8%)	27 (5.8%)	< 0.001	1460 (2.7%)	14 (3.7%)	0.304
Diabetes with complication	9756 (11.2%)	75 (16.9%)	< 0.001	8723 (10.5%)	131 (28.2%)	< 0.001	5777 (10.7%)	106 (28%)	< 0.001
Hemiplegia or paraplegia	487 (0.6%)	2 (0.4%)	1.000	353 (0.4%)	7 (1.5%)	0.001	487 (0.9%)	4 (1.1%)	0.965
Kidney disease	5001 (5.7%)	25 (5.6%)	0.998	2869 (3.5%)	41 (8.8%)	< 0.001	1519 (2.8%)	17 (4.5%)	0.071
Local tumor, leukemia, and lymphoma	31,269 (35.8%)	329 (73.9%)	< 0.001	4304 (5.2%)	44 (9.5%)	< 0.001	1594 (2.9%)	19 (5%)	0.027
Metastatic solid tumor	5516 (6.3%)	87 (19.6%)	< 0.001	812 (1%)	11 (2.4%)	0.005	304 (0.6%)	15 (4%)	< 0.001
Mild liver disease	7694 (8.8%)	55 (12.4%)	0.011	2513 (3%)	32 (6.9%)	< 0.001	1,486 (2.7%)	15 (4%)	0.203
Severe liver disease	1321 (1.5%)	11 (2.5%)	0.146	823 (1%)	24 (5.4%)	< 0.001	266 (0.5%)	5 (1.3%)	0.056

*P-value were calculated for t-test for numerical variable and chi-square test for categorical variable under 0.05 significance. *SD* Standard deviation,

Other Route of arrival contains transfer in, referral from outpatient, other and unknown. Other in Mode of transport contains walk-in,public transportation, Aeromedical transport, other cars, others and unknown.

Based on the variable importance from the Autoscore framework shown in Table 2 and parsimonious plot shown in Supplementary Fig. 1, we selected top 8 variables for score generations. Common feature for three hospitals were vital sign, age, patient consciousness. Vital sign such as systolic blood pressure (SBP) and heart rate (HR) were important in hospital A and B, whereas Consciousness was most important in hospital C. SBP, HR, Temperature were top 3 contributed variables in overall rank.

**Table 2:** Top 14 contribution variables for each hospital.

Top Variable	Hospital A	Hospital B	Hospital C
1	Systolic blood pressure	Heart rate	Consciousness
2	Heart rate	Systolic blood pressure	Systolic blood pressure
3	Temperature	Age	SpO2
4	Diastolic blood pressure	Diastolic blood pressure	Temperature
5	Age	Temperature	Age
6	SpO2	SpO2	Diastolic blood pressure
7	Respiratory rate	Respiratory rate	Heart rate
8	Day of week	Day of week	Respiratory rate
9	KTAS	Consciousness	KTAS
10	Time of visit	Time of visit	Time of visit
11	Consciousness	KTAS	Day of week
12	Route of arrival	Route of arrival	Route of arrival
13	Gender	Gender	Gender
14	Ambulance use	Ambulance use	Ambulance use

*KTAS* Korea Triage Acute Scale.

Scores for each hospital were presented in Table 3. The developed score for each hospital had different patterns. Among the included variables, Temperature and SpO2 were the highest effect in hospital A (17), patient consciousness for hospital B (27) and C (33). In hospital B, Age (13) was also high scored variables. Whereas Systolic blood pressure (14) was dominant at hospital C. Overall score was calculated with weighted score of number of patients and performance for each institutions. Score based on SMOTE was provided at Supplementary Table 4.

**Table 3:** Score generated from each hospital.

Score for 2-day mortality				
Variable	Hospital A	Hospital B	Hospital C	Overall
Age, year				
< 60	0	0	0	0
60–80	4	13	11	8
≥ 80	4	20	12	11
Heart rate, /min				
< 50	4	7	2	5
50–100	0	0	0	0
≥ 100	9	7	2	7
Respiration rate, /min				
< 24	0	0	0	0
≥ 24	13	7	6	10
Temperature, °C				
< 24	17	7	10	12
≥ 24	0	0	0	0
Blood pressure, mm Hg				
Systolic				
< 90	9	7	14	9
≥ 90	0	0	0	0
Diastolic				
< 60	4	7	1	5
≥ 60	0	0	0	0
SpO2, %				
< 90	17	13	14	15
90–95	4	7	5	5
≥ 95	0	0	0	0
Patient consciousness				
Alert	0	0	0	0
Verbal	9	13	12	11
Painful	13	20	19	17
Unconsciousness	13	33	40	24

Variables were selected from parsimonious plot shown in Supplementary Fig. 2. Overall score was calculated with weighted score for each institutions. weights are 0.472 for Hospital A, 0.410 for Hospital B and 0.116 for Hospital C.

We evaluated each score to the other hospital for the intra-institutional external validation. We used the testing cohort to evaluate the performance of each score. Table 4 depicts the AUROC with CI for the external validation which showed the best internal validation (0.913, 0.919 and 0.930) and dropped a little for the external results. Overall evaluation results show the quite good classification results from 0.904 to 0.933. Other metrics for original and SMOTE were shown in Supplementary Table 5.

**Table 4:** Inter-hospital external validation result with AUROC (95% CI mortality) for each hospital.

AUROC (Original)	Validation cohort		
Development cohort	Hospital A	Hospital B	Hospital C
Hospital A	0.913 (0.882–0.945)	0.9124 (0.884–0.9407)	0.928 (0.902–0.955)
Hospital B	0.893 (0.854–0.931)	0.919 (0.891–0.946)	0.930 (0.902–0.958)
Hospital C	0.885 (0.842–0.927)	0.929 (0.9015–0.950)	0.930 (0.899–0.960)
Overall	0.904 (0.866–0.942)	0.929 (0.9049–0.952)	0.933 (0.904–0.961)

*AUROC* area under the receiver operating characteristic.

## Discussion

In this study, we developed interpretable score based on CDM Autoscore for ED and evaluated with 3 tertiary hospitals in Korea for inferring the 2-day mortality for ED visit patients. Although each hospitals have different characteristics, scores were accurate for their external validation results for other institutions which has at least of 0.885 (0.842–0.942) AUROC. Moreover, it was interpretable score, so it can be integrated easily into clinical practice. We found each scores from their own hospital, which is the internal validation results were accurate from 0.913 to 0.930 AUROC. We also identified the extent of lack of accuracy and acceptance when we apply the score to other institute.

To the best of our knowledge, this is the first study for interpretable machine learning using CDM framework in ED. Many policies or laws regarding the data protection or leak was published for the protection of private patient information^18,19^. For solving these problems, our framework can share the result without any transferring patient data. CDM is designed to standardize the structure and vocabulary of observational health data that can produce reliable evidence without sharing data. This approach creates a unique opportunity of implementing several existing data exploration and evidence generation tools and participating in world-wide distributed research network studies without raw data leakage^20–22^. Extensibility and generatability can be obtained based on our framework. More institutions can be added to analysis cohort for further development and validation because of the developed semi-automated ETL process enables CDM conversion for all institution’s NEDIS data in Korea.

Interpretable point-based score can be easily utilized for the real practice. A paper published from Netherlands in 2023 also developed international early warning score for predicting mortality in ED^23^. The score was consistent with our interpretable score in terms of having high impact on consciousness, systolic blood pressure and temperature and Spo2. Whereas old age was most impact factor in international score.

Another novelty for this study is it conducted the cross-external validation for identifying the generalizability. Patient distribution is different for each institution. In case of hospital C, almost mortality patients had severe KTAS level and consciousness was most important for predicting mortality. We need to develop each score for institution. Many previous study emphasized the importance of external validation for the generality of model^14,24,25^. Most of the studies conducted one model from one site to other sites^26,27^, but in this study all institutions made their one score and we can compare the results for each one.

There are some limitations for this study, first it was a retrospective, the score needs to be evaluated in prospectively for the checking the applicability. However, this score-based model development is easy to apply to EMR integration because of advantages of point-based score. Second, we need to consider the representative score for Korea. We can develop with national emergency department information system data which is data from 403 ED data for developing national level score for Korea.

In summary, we developed the K-SERP score for 3 hospitals in Korea using CDM Autoscore for ED and showed good cross-external validation results which were at least 0.899 of AUROC. We can expand the result with other emergency department site based on CDM framework. Each score could be interpreted and applied to clinical process easily.

## Method

### Study design and setting

This retrospective and validation study was executed across from 3 ED in Korea (A, B and C). A, B and C are tertiary hospitals located in a metropolitan city in Korea. Respectively, the hospital has approximately 2000, 1000, and 1000 inpatient beds. Approximately more than 80,000, 90,000 and 50,000 patients visit the ED annually. There are 16, 20 and 7 specialists working at each institution, respectively. All data were mapped to the Observational Medical Outcome Partnership Common Data Model (OMOP-CDM) for the multicenter study. This study was approved by the Samsung Medical Center Institutional Review Board (2023-02-036), and a waiver of informed consent was granted for EHR data collection and analysis because of the retrospective and de-identified nature of the data. All methods were performed in accordance with the relevant guidelines and regulations.

### Selection of participants

Initially, ED patients from 2016 to 2017 were included for each hospital. Patient older than 18 with disease patients were included. We also excluded patient with left without being seen or death on arrival/cardiopulmonary resuscitation patients. We split into two cohort: development (70%) cohort for training the interpretable ML model and test (30%) for evaluation from each hospital.

### Candidate predictors

We extracted data from each hospital’s electronic medical records system which all patient information was deidentified. Candidate input variables were considered with available features at the stage of ED triage including demographic characteristics such as age, gender, administrative variables including time of ED visit and clinical variables such as severity index, consciousness, and initial vital sign. Comorbidities were also obtained from hospital diagnosis records in the preceding 5 years before patients’ emergency visit and compared for each hospital. They were extracted from International Statistical Classification of Diseases and Related Health Problems, Tenth Revision (ICD-10). The list and description of candidate predictors and comorbidities are given in the supplementary Tables 6 and 7.

### Outcomes

Emergency patients with semi-acute conditions typically undergo surgical procedure or are admitted to Intensive care unit (ICU) following emergency room treatment and given the imperative for patients to survive. Our primary outcome was 2-day mortality which was the target feature for analysis to build the interpretable ML model for each hospital.

### Common data model (CDM)

**Figure 2:**
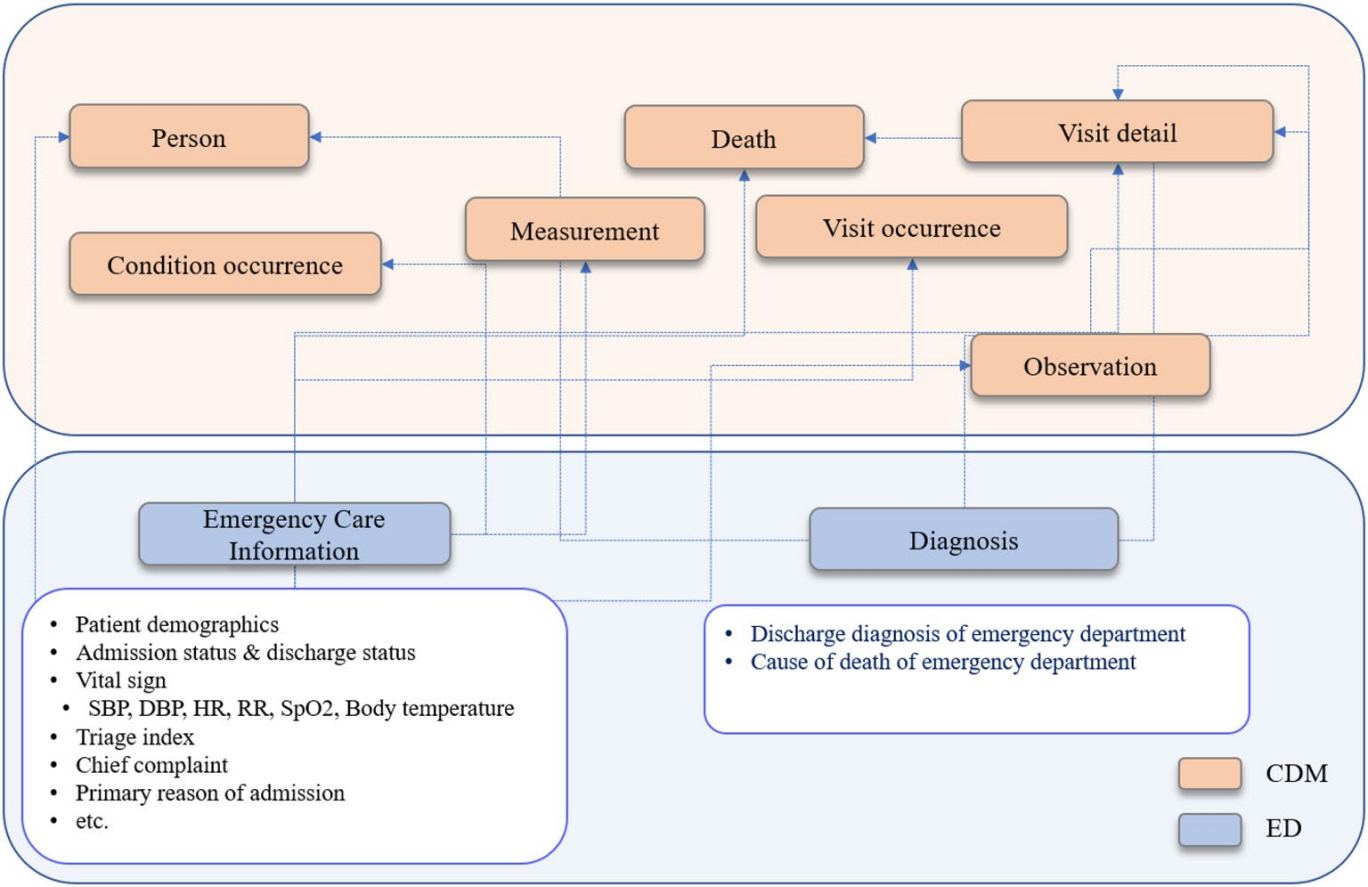
Table mapping for converting clinical to common data model tables. CDM: common data model; ED: Emergency department.

For the multicenter study, we adopted OMOP CDM from the research network Observational Health Data Sciences and Informatics (OHDSI)^28^ for standardized structure and vocabularies to map emergency department data based on Systematized Nomenclature of Medicine–Clinical Terms (SNOMED-CT) and Logical Observation Identifiers Names and Codes (LOINC) as example shown Supplementary Fig. 1. Extract, Transformation and Load (ETL) process was performed with structured query language. Each ED care and diagnosis related information was mapped into proper CDM tables as shown in Fig. 2. For example, patient demographics and vital sign are mapped to Person and Measurement table, respectively. After transformation was completed into CDM format, all hospital can get the same structure and vocabularies, for executing same research query. All details of transformation and code are accessible on Gitgub^29^.

### CDM autoscore for ED framework

AutoScore Framework is a machine learning-based clinical score generator, consisting of six modules developed from Singapore^12^. Module 1 uses a random forest for ranking variables according to their importance. Module 2 transforms variables by categorizing continuous variables to improve interpretation with quantile information. Module 3 makes scores for each variable based on a logistic regression coefficient. Module 4 selects which variables could be included in the scoring model. In Module 5, clinical domain knowledge is incorporated to the score and cutoff points can be defined when categorizing continuous variables. Module 6 evaluates the performance of the score in a separate test dataset. The AutoScore framework provides a systematic and automated approach to develop score automatically, combining of advantage of machine learning for discriminating and the strength of logistic regression in its interpretability. For the overall score generation, We considered weighted average scores across all institutions. For each institutions *i*, a weight ${w}_{i}$ was formulated as ${w}_{i}$ = $\left(\sqrt{{(AUC}_{i})} \times {N}_{i}^{3}\right)$/${\sum }_{i=1}^{M}\sqrt{{(AUC}_{i})} \times {N}_{i}^{3})$ × 100% where ${N}_{i}$ was the sample size, ${AUC}_{i}$ was the AUC value obtained based on the validation set, and *M* was the total number of institutions. Overall score was calculated with weighted score based on ${w}_{i}$.

**Figure 3:**
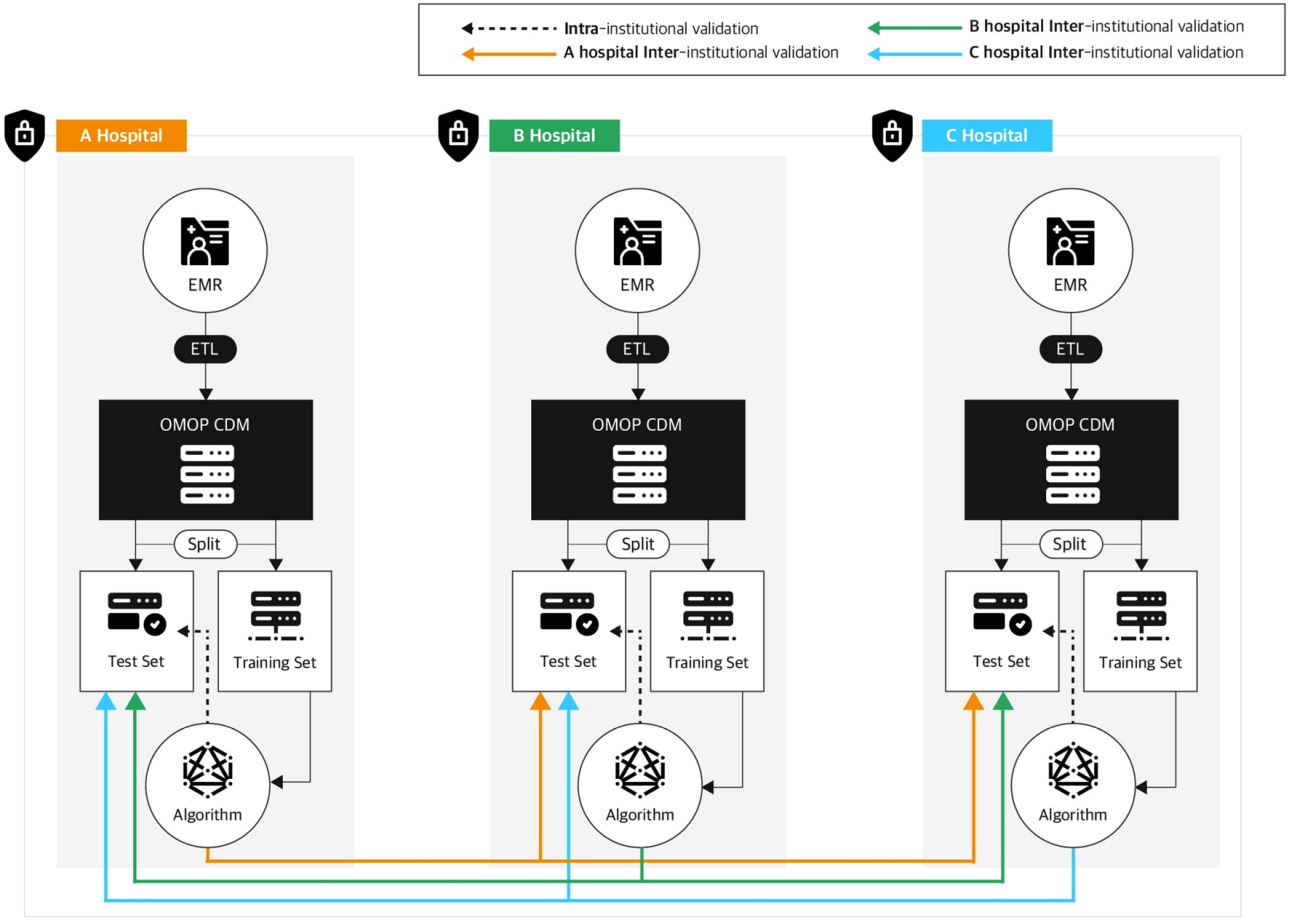
Overall process of “CDM Autoscore for ED”. Each Institutions conducted Extract, Transformation and Load process for converting local data into CDM format. Algorithms from each of institution were derived using interpretable machine learning framework and validated inter-and intra- institutionally. EMR: Electronic medical records; ETL: Extract, transformation and Load; OMOP CDM: Observational Medical Outcome Partnership Common Data Model.

We defined our new novel framework “CDM Autoscore for ED”, combination of CDM based standardized format and autoscore based interpretable framework shown in Fig. 3. The analysis and preparation code using CDM format was also shared on GitHub^29^.

### Statistical analysis

Categorical features were expressed as frequency and percentages and continuous features were expressed as means and standard deviations. Comparison tests for each hospital were performed with analysis of variance and chi-square tests at 5% significance levels. Standardized mean difference (SMD) was also calculated for comparing each hospital. Two types of validations for this study were conducted. First, we executed internal-institutional validation for each hospital’s score. We also performed intra-institutional validation pair-wisely for the external validation. Area under the curve in the receiver operating characteristic (AUROC) and 95% confidence interval (CI) with 1000 times of bootstrap was reported. Other metrics including accuracy, sensitivity, specificity, positive predictive value (PPV) and negative predictive value (NPV) were also reported. SMOTE was conducted for handling the imbalance problem. Twice of minority was oversampled and same number of majorities according to the number of minority was sampled with fixed seed number.

### Supplementary Information

Supplementary Information.

## Data Availability

Data was available in study site clinical data warehouse. The datasets generated and analyzed during the current study are not publicly available due dataset includes although is de-identifed, part of patient information, but are available from the corresponding author on reasonable request.

## Abbreviations

ED: Emergency department
ML: Machine learning
SERP: Score for emergency risk prediction
CDM: Common data model
AUROC: Area under receiver operating curve
KTAS: Korea triage acuity scale
SMOTE: Synthetic minority over-sampling technique
SMD: Standardized mean difference
OMOP-CDM: Observational medical outcome partnership common data model
OHDSI: Observational health data sciences and informatics
SNOMED-CT: Systematized nomenclature of medicine–clinical terms
LOINC: Logical observation identifiers names and codes
ETL: Extract transformation load
EMR: Electronic medical records
CI: Confidence interval
SD: Standard deviation
