# Supplementary material for: Inter hospital external validation of interpretable machine learning based triage score for the emergency department using common data model
Source: Sci Rep. 2024 Mar 20;14:6666. doi: 10.1038/s41598-024-54364-7 (PMC10954621; doi:10.1038/s41598-024-54364-7)
Supplement: Supplementary file 1 — Supplementary Information. [file 41598_2024_54364_MOESM1_ESM.docx]

Supplementary


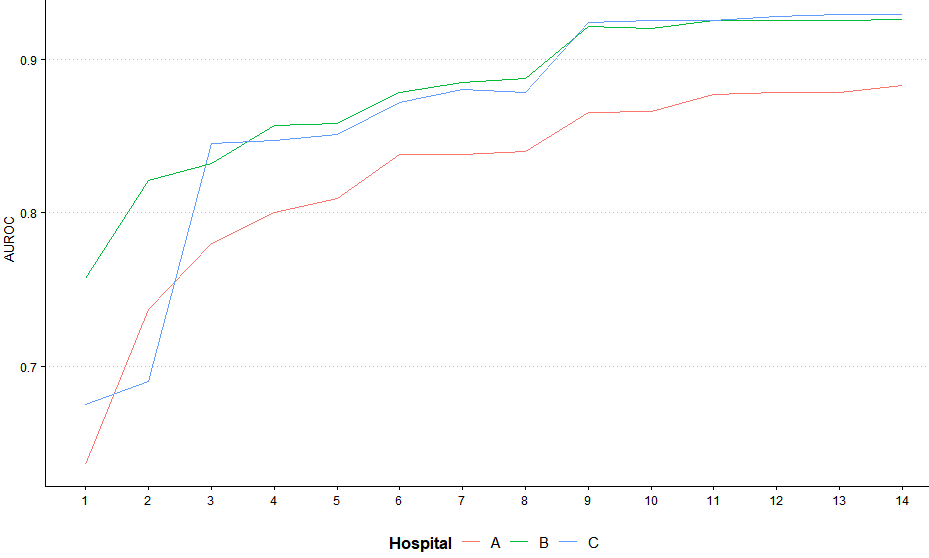


**Supplementary Figure1. Parsimonious plot for each hospital. x axis means number of input variable for modeling. Y axis means area under the receiver operating characteristic curve (AUROC). Order of input variable was determined by feature importance from random forest algorithm. Number of needed variables was selected when there is no significant increase of AUROC.**


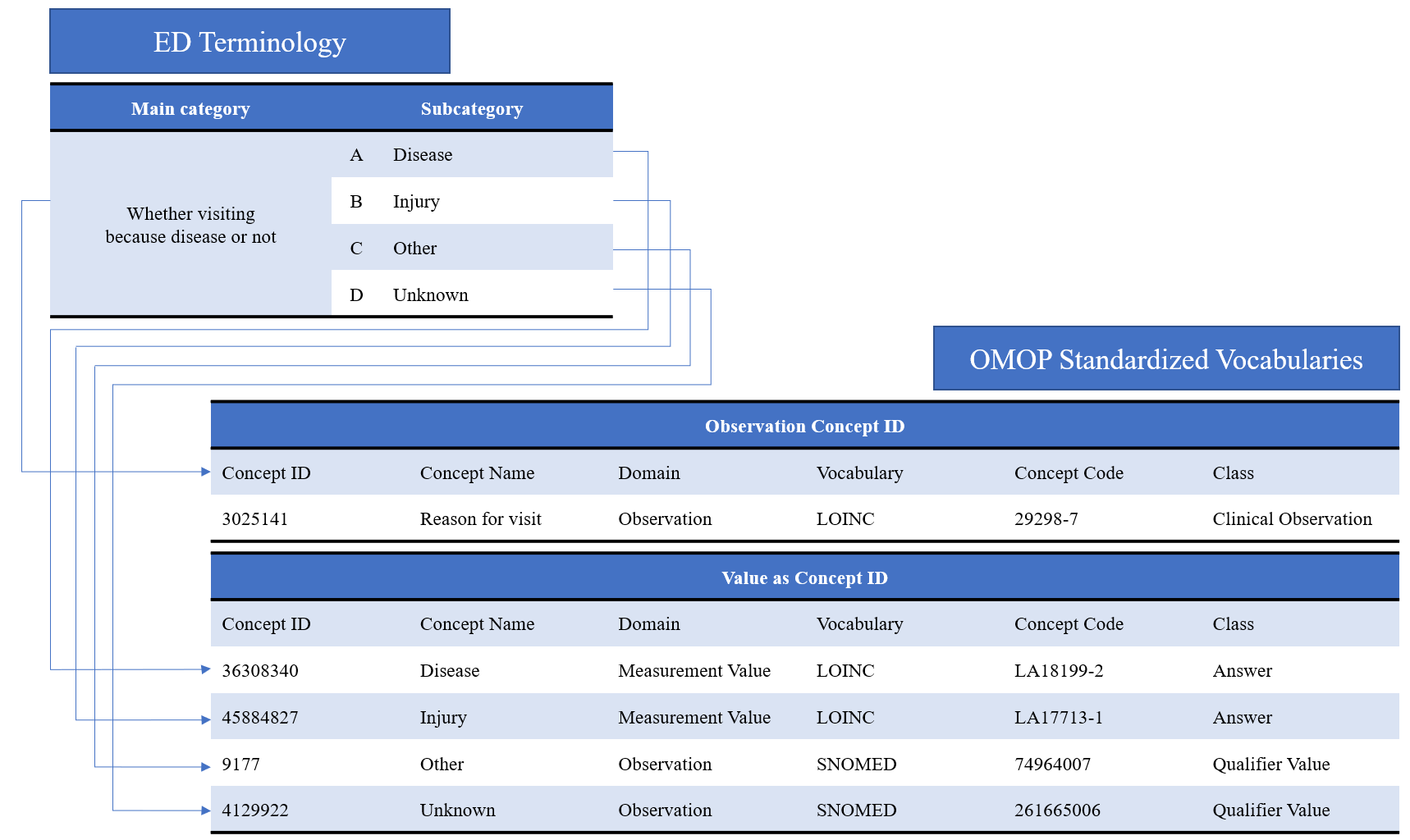


**Supplementary Figure 2. Terminology mapping to Observational Medical Outcomes Partnership standardized vocabulary.**


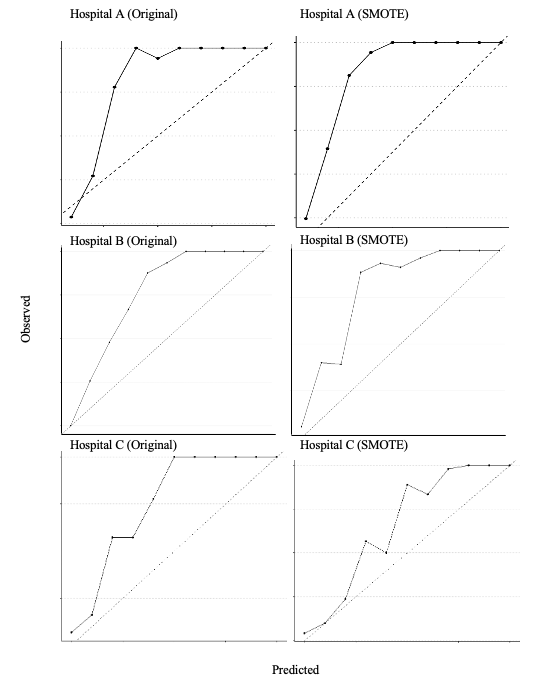


**Supplementary Figure 3.** calibration between observed and predicted 2 day mortality for each hospitals with and without data imbalance strategy.

SMOTE : Synthetic minority over-sampling technique

**Supplementary Table1.** Baseline Demographic for each hospital ED triage information from 2016 to 2017 using SMOTE**.**

| **SMOTE** | **Hospital A** | | | **Hospital B** | | | **Hospital C** | | | |
| --- | --- | --- | --- | --- | --- | --- | --- | --- | --- | --- |
|  | **No death**  **(n=890)** | **2 d- mortality**  **(n=890)** | **p-value** | **No death**  **(n=928)** | **2 d- mortality**  **(n=928)** | **p-value** | | **No death**  **(n=758)** | **2 d- mortality**  **(n=758)** | **p-value** |
| Sex |  |  | <0.001 |  |  | 0.019 | |  |  | <0.001 |
| Male | 449 (50.4%) | 534 (60.0%) |  | 425 (45.8%) | 519 (55.9%) |  | | 341 (45.0%) | 441 (58.2%) |  |
| Female | 441 (49.6%) | 356 (40.0%) |  | 503 (54.2%) | 409 (44.1%) |  | | 417 (55.0%) | 317 (41.8%) |  |
| Age, mean (SD) | 55.7 ± 17.4 | 67.4 ± 13.2 | <0.001 | 50.5 ± 18.6 | 72.6 ± 12.7 | <0.001 | | 51.6 ± 20.5 | 71.1 ± 14.3 | <0.001 |
| Day of week |  |  | 0.002 |  |  | <0.001 | |  |  | 0.026 |
| Midweek | 358 (40.1%) | 382 (43.0%) |  | 376 (40.5%) | 279 (30.1%) |  | | 284 (37.5%) | 298 (39.3%) |  |
| Weekend | 236 (26.6%) | 260 (29.2%) |  | 265 (28.6%) | 299 (32.2%) |  | | 251 (33.1%) | 239 (31.5%) |  |
| Friday | 136 (15.3%) | 91 (10.2%) |  | 135 (14.5%) | 132 (14.2%) |  | | 102 (13.5%) | 71 (9.4%) |  |
| Monday | 160 (18.0%) | 157 (17.6%) |  | 152 (16.4%) | 218 (23.5%) |  | | 121 (16.0%) | 150 (19.8%) |  |
| Shift time |  |  | 0.002 |  |  | 0.114 | |  |  | 0.032 |
| 8 am to 4 pm | 409 (46.0%) | 443 (49.8%) |  | 406 (43.8%) | 390 (42.0%) |  | | 274 (36.1%) | 284 (37.5%) |  |
| 4 pm to midnight | 327 (36.7%) | 254 (28.5%) |  | 336 (36.2%) | 315 (33.9%) |  | | 298 (39.3%) | 253 (33.4%) |  |
| Midnight to 8 am | 154 (17.3%) | 193 (21.7%) |  | 186 (20.0%) | 223 (24.0%) |  | | 186 (24.5%) | 221 (29.2%) |  |
| Triage Categories |  |  | <0.001 |  |  | <0.001 | |  |  | <0.001 |
| 1 (most severe) | 5 (0.6%) | 138 (15.5%) |  | 12 (1.3%) | 216 (23.3%) |  | | 11 (1.5%) | 385 (50.8%) |  |
| 2 | 93 (10.4%) | 282 (31.7%) |  | 98 (10.6%) | 370 (39.9%) |  | | 92 (12.1%) | 271 (35.8%) |  |
| 3 | 412 (46.3%) | 384 (43.1%) |  | 594 (64.0%) | 334 (36.0%) |  | | 456 (60.2%) | 100 (13.2%) |  |
| 4 | 308 (34.6%) | 83 (9.3%) |  | 172 (18.5%) | 6 (0.6%) |  | | 153 (20.2%) | 2 (0.3%) |  |
| 5 (less severe) | 72 (8.1%) | 3 (0.3%) |  | 52 (5.6%) | 2 (0.2%) |  | | 46 (6.1%) | 0 (0.0%) |  |
| Consciousness |  |  | <0.001 |  |  | <0.001 | |  |  | <0.001 |
| Alert | 874 (98.2%) | 641 (72.0%) |  | 883 (95.2%) | 439 (47.3%) |  | | 713 (94.1%) | 228 (30.1%) |  |
| Verbal | 8 (0.9%) | 106 (11.9%) |  | 23 (2.5%) | 174 (18.8%) |  | | 37 (4.9%) | 101 (13.3%) |  |
| Painful | 8 (0.9%) | 102 (11.5%) |  | 17 (1.8%) | 182 (19.6%) |  | | 8 (1.1%) | 187 (24.7%) |  |
| Unconsciousness | 0 (0.0%) | 41 (4.6%) |  | 5 (0.5%) | 133 (14.3%) |  | | 0 (0.0%) | 242 (31.9%) |  |
| Route of arrival |  |  | <0.001 |  |  | <0.001 | |  |  | 0.012 |
| Direct | 699 (78.5%) | 563 (63.3%) |  | 722 (77.8%) | 409 (44.1%) |  | | 638 (84.2%) | 599 (79.0%) |  |
| Other* | 191 (21.5%) | 327 (36.7%) |  | 206 (22.2%) | 519 (55.9%) |  | | 120 (15.8%) | 159 (21.0%) |  |
| Mode of transport |  |  | <0.001 |  |  | <0.001 | |  |  | <0.001 |
| Ambulance | 168 (18.9%) | 641 (72.0%) |  | 193 (20.8%) | 717 (77.3%) |  | | 225 (29.7%) | 685 (90.4%) |  |
| Other* | 722 (81.1%) | 249 (28.0%) |  | 735 (79.2%) | 211 (22.7%) |  | | 533 (70.3%) | 73 (9.6%) |  |
| Vital signs, mean (SD) |  |  |  |  |  |  | |  |  |  |
| Pulse, /min | 90.4 ± 21.1 | 108.3 ± 23.3 | <0.001 | 87.3 ± 17.6 | 98.5 ± 24.7 | <0.001 | | 88.3 ± 19.4 | 97.8 ± 18.8 | <0.001 |
| Blood pressure, mm Hg |  |  |  |  |  |  | |  |  |  |
| Systolic | 129.8 ± 25.6 | 117.5 ± 29.7 | <0.001 | 131.6 ± 24.9 | 108.0 ± 29.3 | <0.001 | | 132.8 ± 25.3 | 114.1 ± 30.5 | <0.001 |
| Diastolic | 77.3 ± 15.2 | 67.8 ± 18.2 | <0.001 | 79.1 ± 15.1 | 62.6 ± 17.5 | <0.001 | | 81.0 ± 15.6 | 72.5 ± 19.3 | <0.001 |
| Respiration, /min | 19.0 ± 2.4 | 22.6 ± 4.9 | <0.001 | 16.2 ± 3.1 | 21.9 ± 5.4 | <0.001 | | 20.9 ± 3.0 | 22.0 ± 6.1 | <0.001 |
| SPo2, % | 97.2 ± 2.7 | 92.0 ± 9.2 | <0.001 | 98.3 ± 2.2 | 94.0 ± 6.6 | <0.001 | | 97.6 ± 3.0 | 90.7 ± 10.1 | <0.001 |
| Temperature, ⁰c | 37.0 ± 0.8 | 36.8 ± 1.0 | <0.001 | 36.8 ± 0.7 | 36.6 ± 1.0 | 0.006 | | 36.8 ± 0.8 | 36.0 ± 1.0 | <0.001 |
| Comorbidity |  |  |  |  |  |  | |  |  |  |
| Myocardial infarction | 19 (2.1%) | 28 (3.1%) | 0.237 | 13 (1.4%) | 73 (7.9%) | <0.001 | | 24 (3.2%) | 35 (4.6%) | 0.184 |
| Congestive heart failure | 62 (7%) | 62 (7%) | 1.000 | 23 (2.5%) | 108 (11.6%) | <0.001 | | 33 (4.4%) | 31 (4.1%) | 0.898 |
| Peripheral vascular disease | 28 (3.1%) | 24 (2.7%) | 0.673 | 11 (1.2%) | 16 (1.7%) | 0.438 | | 3 (0.4%) | 7 (0.9%) | 0.341 |
| Stroke | 93 (10.4%) | 80 (9%) | 0.337 | 61 (6.6%) | 111 (12%) | <0.001 | | 33 (4.4%) | 36 (4.7%) | 0.805 |
| Dementia | 31 (3.5%) | 42 (4.7%) | 0.232 | 15 (1.6%) | 23 (2.5%) | 0.251 | | 13 (1.7%) | 38 (5%) | 0.001 |
| Chronic pulmonary disease | 55 (6.2%) | 101 (11.3%) | <0.001 | 47 (5.1%) | 95 (10.2%) | <0.001 | | 23 (3%) | 68 (9%) | <0.001 |
| Rheumatoid disease | 12 (1.3%) | 9 (1%) | 0.661 | 8 (0.9%) | 9 (1%) | 1 | | 7 (0%) | 6 (0.8%) | 1 |
| Diabetes without complications | 36 (4%) | 29 (3.3%) | 0.448 | 25 (2.7%) | 86 (9.3%) | <0.001 | | 25 (3.3%) | 21 (2.9%) | 0.653 |
| Diabetes with complication | 112 (12.6%) | 136 (15.3%) | 0.115 | 106 (11.4%) | 289 (31.1%) | <0.001 | | 94 (12.4%) | 218 (28.8%) | <0.001 |
| Hemiplegia or paraplegia | 6 (0.7%) | 4 (0.4%) | 0.751 | 3 (0.3%) | 12 (1.3%) | 0.038 | | 10 (1.3%) | 7 (0.9%) | 0.626 |
| Kidney disease | 54 (6.1%) | 45 (5.1%) | 0.408 | 30 (3.2%) | 100 (10.8%) | <0.001 | | 27 (3.6%) | 24 (3.2%) | 0.776 |
| Local tumor, leukemia, and lymphoma | 330 (37.1%) | 667 (74.9%) | <0.001 | 47 (5.1%) | 113 (12.2%) | <0.001 | | 16 (2.1%) | 83 (10.9%) | <0.001 |
| Metastatic solid tumor | 60 (6.7%) | 171 (19.2%) | <0.001 | 12 (1.3%) | 14 (1.5%) | 0.843 | | 6 (0.8%) | 24 (3.2%) | 0.002 |
| Mild liver disease | 66 (7.4%) | 93 (10.4%) | 0.031 | 40 (4.3%) | 51 (5.5%) | 0.282 | | 24 (3.2%) | 24 (3.2%) | 1 |
| Severe liver disease | 12 (1.3%) | 22 (2.5%) | 0.119 | 11 (1.2%) | 41 (4.4%) | <0.001 | | 5 (0.7%) | 6 (0.8%) | 1 |

SMOTE : Synthetic minority over-sampling technique

[Changes in Results]

**Supplementary Table2.** Standarzied mean difference (SMD) for each hospital ED triage information

| Original | No death | | | 2d- mortality | | |
| --- | --- | --- | --- | --- | --- | --- |
| SMD | A vs B | A vs C | B vs C | A vs B | A vs C | B vs C |
| Sex | 0.065 | 0.103 | 0.038 | 0.205 | 0.284 | 0.077 |
| Age, mean (SD) | 0.003 | 0.003 | 0.000 | 0.070 | 0.066 | 0.004 |
| Day of week |  |  |  |  |  |  |
| Midweek | 0.022 | 0.028 | 0.007 | 0.032 | 0.013 | 0.018 |
| Weekend | 0.041 | 0.059 | 0.018 | 0.000 | 0.055 | 0.055 |
| Friday | 0.005 | 0.007 | 0.002 | 0.054 | 0.054 | 0.000 |
| Monday | 0.018 | 0.027 | 0.009 | 0.004 | 0.041 | 0.046 |
| Shift time |  |  |  |  |  |  |
| 8 am to 4 pm | 0.061 | 0.132 | 0.071 | 0.097 | 0.098 | 0.002 |
| 4 pm to midnight | 0.005 | 0.044 | 0.039 | 0.186 | 0.089 | 0.095 |
| Midnight to 8 am | 0.071 | 0.112 | 0.040 | 0.095 | 0.020 | 0.118 |
| Triage Categories |  |  |  |  |  |  |
| 1 (most severe) | 0.000 | 0.055 | 0.055 | 0.037 | 0.696 | 0.649 |
| 2 | 0.042 | 0.12 | 0.077 | 0.338 | 0.036 | 0.295 |
| 3 | 0.353 | 0.271 | 0.081 | 0.213 | 0.512 | 0.305 |
| 4 | 0.371 | 0.354 | 0.018 | 0.298 | 0.314 | 0.037 |
| 5 (less severe) | 0.077 | 0.071 | 0.007 | 0.000 | 0.063 | 0.063 |
| Consciousness |  |  |  |  |  |  |
| Alert | 0.084 | 0.136 | 0.05 | 0.467 | 0.768 | 0.268 |
| Verbal | 0.055 | 0.145 | 0.086 | 0.186 | 0.161 | 0.023 |
| Painful | 0.068 | 0.032 | 0.032 | 0.304 | 0.107 | 0.18 |
| Unconsciousness | 0.000 | 0.000 | 0.000 | 0.165 | 0.768 | 0.572 |
| Route of arrival | 0.028 | 0.114 | 0.141 | 0.281 | 0.227 | 0.51 |
| Mode of transport | 0.016 | 0.193 | 0.176 | 0.211 | 0.446 | 0.242 |
| Vital signs, mean (SD) |  |  |  |  |  |  |
| Pulse, /min | 0.001 | 0.023 | 0.001 | 0.055 | 0.068 | 0.012 |
| Blood pressure, mm Hg |  |  |  |  |  |  |
| Systolic | 0.001 | 0.034 | 0.002 | 0.103 | 0.045 | 0.058 |
| Diastolic | 0.001 | 0.027 | 0.003 | 0.054 | 0.055 | 0.107 |
| Respiration, /min | 0.006 | 0.013 | 0.009 | 0.014 | 0.014 | 0.000 |
| SPo2, % | 0.002 | 0.033 | 0.002 | 0.023 | 0.044 | 0.072 |
| Temperature, ⁰c | 0.001 | 0.020 | 0.000 | 0.009 | 0.033 | 0.021 |
| Comorbidity |  |  |  |  |  |  |
| Myocardial infarction | 0.000 | 0.032 | 0.032 | 0.046 | 0.099 | 0.052 |
| Congestive heart failure | 0.102 | 0.102 | 0.000 | 0.046 | 0.093 | 0.135 |
| Peripheral vascular disease | 0.097 | 0.090 | 0.009 | 0.034 | 0.094 | 0.064 |
| Stroke | 0.091 | 0.118 | 0.029 | 0.013 | 0.127 | 0.115 |
| Dementia | 0.114 | 0.048 | 0.081 | 0.098 | 0.027 | 0.137 |
| Chronic pulmonary disease | 0.081 | 0.130 | 0.053 | 0.104 | 0.194 | 0.097 |
| Rheumatoid disease | 0.022 | 0.047 | 0.026 | 0.000 | 0.025 | 0.025 |
| Diabetes without complications | 0.049 | 0.053 | 0.005 | 0.070 | 0.013 | 0.078 |
| Diabetes with complication | 0.018 | 0.013 | 0.005 | 0.230 | 0.226 | 0.004 |
| Hemiplegia or paraplegia | 0.022 | 0.029 | 0.054 | 0.103 | 0.072 | 0.028 |
| Kidney disease | 0.083 | 0.112 | 0.032 | 0.105 | 0.040 | 0.135 |
| Local tumor, leukemia, and lymphoma | 0.607 | 0.666 | 0.091 | 1.326 | 1.480 | 0.136 |
| Metastatic solid tumor | 0.210 | 0.229 | 0.035 | 0.418 | 0.371 | 0.077 |
| Mild liver disease | 0.188 | 0.200 | 0.015 | 0.147 | 0.235 | 0.100 |
| Severe liver disease | 0.036 | 0.076 | 0.045 | 0.130 | 0.068 | 0.171 |

SMD : Standardized mean difference

**Supplementary Table3.** Standarzied mean difference (SMD) for each hospital ED triage information using SMOTE.

| SMOTE | No death | | | 2d- Mortality | | |
| --- | --- | --- | --- | --- | --- | --- |
| SMD | A vs B | A vs C | B vs C | A vs B | A vs C | B vs C |
| Sex | 0.075 | 0.088 | 0.013 | 0.264 | 0.303 | 0.038 |
| Age, mean (SD) | 0.042 | 0.033 | 0.008 | 0.048 | 0.034 | 0.014 |
| Day of week |  |  |  |  |  |  |
| Midweek | 0.007 | 0.043 | 0.050 | 0.218 | 0.061 | 0.160 |
| Weekend | 0.037 | 0.118 | 0.080 | 0.053 | 0.041 | 0.012 |
| Friday | 0.375 | 0.042 | 0.447 | 0.102 | 0.022 | 0.118 |
| Monday | 0.034 | 0.043 | 0.009 | 0.122 | 0.046 | 0.073 |
| Shift time |  |  |  |  |  |  |
| 8 am to 4 pm | 0.036 | 0.164 | 0.128 | 0.128 | 0.203 | 0.075 |
| 4 pm to midnight | 0.008 | 0.044 | 0.052 | 0.096 | 0.087 | 0.009 |
| Midnight to 8 am | 0.057 | 0.148 | 0.090 | 0.045 | 0.143 | 0.097 |
| Triage Categories |  |  |  |  |  |  |
| 1 (most severe) | 0.063 | 0.078 | 0.014 | 0.166 | 0.698 | 0.499 |
| 2 | 0.005 | 0.044 | 0.039 | 0.141 | 0.071 | 0.069 |
| 3 | 0.293 | 0.229 | 0.064 | 0.118 | 0.544 | 0.425 |
| 4 | 0.293 | 0.260 | 0.035 | 0.294 | 0.307 | 0.035 |
| 5 (less severe) | 0.079 | 0.062 | 0.018 | 0.016 | 0.055 | 0.045 |
| Consciousness |  |  |  |  |  |  |
| Alert | 0.149 | 0.192 | 0.041 | 0.432 | 0.756 | 0.289 |
| Verbal | 0.110 | 0.223 | 0.110 | 0.162 | 0.035 | 0.120 |
| Painful | 0.068 | 0.017 | 0.046 | 0.191 | 0.299 | 0.102 |
| Unconsciousness | 0.100 | 0.000 | 0.071 | 0.299 | 0.699 | 0.366 |
| Route of arrival | 0.014 | 0.118 | 0.131 | 0.322 | 0.280 | 0.608 |
| Mode of transport | 0.039 | 0.205 | 0.164 | 0.099 | 0.131 | 0.033 |
| Vital signs, mean (SD) |  |  |  |  |  |  |
| Pulse, /min | 0.023 | 0.217 | 0.008 | 0.068 | 0.073 | 0.005 |
| Blood pressure, mm Hg |  |  |  |  |  |  |
| Systolic | 0.012 | 0.332 | 0.008 | 0.058 | 0.021 | 0.037 |
| Diastolic | 0.015 | 0.268 | 0.016 | 0.041 | 0.037 | 0.078 |
| Respiration, /min | 0.061 | 0.131 | 0.088 | 0.011 | 0.009 | 0.001 |
| SPo2, % | 0.022 | 0.332 | 0.015 | 0.022 | 0.014 | 0.042 |
| Temperature, ⁰c | 0.007 | 0.199 | 0.000 | 0.007 | 0.027 | 0.020 |
| Comorbidity |  |  |  |  |  |  |
| Myocardial infarction | 0.042 | 0.058 | 0.105 | 0.186 | 0.066 | 0.107 |
| Congestive heart failure | 0.162 | 0.089 | 0.089 | 0.135 | 0.100 | 0.215 |
| Peripheral vascular disease | 0.100 | 0.151 | 0.068 | 0.054 | 0.103 | 0.055 |
| Stroke | 0.108 | 0.178 | 0.077 | 0.082 | 0.133 | 0.204 |
| Dementia | 0.093 | 0.088 | 0.006 | 0.092 | 0.011 | 0.114 |
| Chronic pulmonary disease | 0.038 | 0.119 | 0.084 | 0.029 | 0.061 | 0.033 |
| Rheumatoid disease | 0.030 | 0.115 | 0.095 | 0.000 | 0.017 | 0.017 |
| Diabetes without complications | 0.057 | 0.030 | 0.029 | 0.220 | 0.019 | 0.204 |
| Diabetes with complication | 0.030 | 0.005 | 0.025 | 0.325 | 0.280 | 0.041 |
| Hemiplegia or paraplegia | 0.044 | 0.052 | 0.103 | 0.088 | 0.054 | 0.030 |
| Kidney disease | 0.108 | 0.092 | 0.018 | 0.183 | 0.075 | 0.227 |
| Local tumor, leukemia, and lymphoma | 0.631 | 0.709 | 0.124 | 1.276 | 1.316 | 0.033 |
| Metastatic solid tumor | 0.206 | 0.229 | 0.039 | 0.439 | 0.387 | 0.098 |
| Mild liver disease | 0.104 | 0.145 | 0.046 | 0.142 | 0.218 | 0.089 |
| Severe liver disease | 0.007 | 0.047 | 0.040 | 0.089 | 0.101 | 0.168 |

SMD : Standardized mean difference ; SMOTE : Synthetic minority over-sampling technique

Supplementary Table4. Score generated from each hospital.

| **SMOTE Score for 2- day Mortality** | | | |  |
| --- | --- | --- | --- | --- |
| **Variable** | Hospital A | Hospital B | Hospital C | Overall |
| Age, year |  |  |  |  |
| < 60 | 0 | 0 | 0 | 0 |
| 60 - 80 | 2 | 12 | 11 | 8 |
| ≥ 80 | 4 | 18 | 14 | 12 |
| Heart rate, /min |  |  |  |  |
| < 50 | 2 | 6 | 0 | 3 |
| 50 - 100 | 0 | 0 | 7 | 2 |
| ≥ 100 | 4 | 6 | 14 | 7 |
| Respiration rate, /min |  |  |  |  |
| < 24 | 0 | 0 | 0 | 0 |
| ≥ 24 | 7 | 6 | 2 | 5 |
| Temperature, ⁰c |  |  |  |  |
| < 24 | 9 | 12 | 14 | 11 |
| ≥ 24 | 0 | 0 | 0 | 0 |
| Blood pressure, mm Hg |  |  |  |  |
| Systolic |  |  |  |  |
| < 90 | 5 | 18 | 11 | 12 |
| ≥ 90 | 0 | 0 | 0 | 0 |
| Diastolic |  |  |  |  |
| < 60 | 4 | 6 | 7 | 6 |
| ≥ 60 | 0 | 0 | 0 | 0 |
| SpO2, % |  |  |  |  |
| <90 | 9 | 18 | 14 | 14 |
| 90 – 95 | 5 | 12 | 4 | 8 |
| ≥ 95 | 0 | 0 | 0 | 0 |
| Patient Consciousness |  |  |  |  |
| Alert | 0 | 0 | 0 | 0 |
| Verbal | 5 | 12 | 9 | 9 |
| Painful | 7 | 12 | 18 | 12 |
| Unconsciousness | 59 | 18 | 23 | 34 |

SMOTE : Synthetic minority over-sampling technique; Overall score was calculated with weighted score for each institutions. weights are 0.356 for Hospital A, 0.417 for Hospital B and 0.225 for Hospital C

Supplementary Table5. Metrics of original and SMOTE from each hospital.

| AUROC (Original) | Validation Cohort | | |
| --- | --- | --- | --- |
| Development Cohort | Hospital A | Hospital B | Hospital C |
| Hospital A | 0.913 (0.882 - 0.945) | 0.9124 (0.884 - 0.9407) | 0.928 (0.902 - 0.955) |
| Hospital B | 0.893 (0.854 - 0.931) | 0.919 (0.891 - 0.946) | 0.930 (0.902 - 0.958) |
| Hospital C | 0.885 (0.842 - 0.927) | 0.929 (0.9015 - 0.950) | 0.930 (0.899 - 0.960) |
| Overall | 0.904 (0.866 - 0.942) | 0.929 (0.9049 - 0.952) | 0.933 (0.904 - 0.961) |

| AUROC (SMOTE) | Validation Cohort | | |
| --- | --- | --- | --- |
| Development Cohort | Hospital A | Hospital B | Hospital C |
| Hospital A | 0.889 (0.855 - 0.923) | 0.921 (0.895 - 0.948) | 0.922 (0.891 - 0.953) |
| Hospital B | 0.856 (0.817 - 0.895) | 0.927 (0.902 - 0.952) | 0.927 (0.898 - 0.957) |
| Hospital C | 0.855 (0.817 - 0.894) | 0.920 (0.894 - 0.947) | 0.931 (0.903 - 0.959) |
| Overall | 0.865 (0.827 - 0.902) | 0.928 (0.903 - 0.953) | 0.931 (0.903 - 0.960) |

| Accuracy (Original) | | Validation Cohort | | | | | |
| --- | --- | --- | --- | --- | --- | --- | --- |
| Development Cohort | | Hospital A | | Hospital B | | Hospital C | |
| Hospital A | | 0.795 (0.787 - 0.909) | | 0.852 (0.693 - 0.953) | | 0.899 (0.768 - 0.942) | |
| Hospital B | | 0.721 (0.712 - 0.897) | | 0.807 (0.801 - 0.813) | | 0.877 (0.736 - 0.962) | |
| Hospital C | | 0.897 (0.798 - 0.920) | | 0.859 (0.798 - 0.927) | | 0.815 (0.808 - 0.823) | |
| Overall | | 0.812 (0.715 - 0.915) | | 0.842 (0.777 - 0.942) | | 0.909 (0.782.- 0.933) | |
| Accuracy (SMOTE) | | Validation Cohort | | | | | |
| Development Cohort | | Hospital A | | Hospital B | | Hospital C | |
| Hospital A | | 0.812 (0.769 - 0.848) | | 0.862 (0.827 - 0.897) | | 0.864 (0.828 - 0.897) | |
| Hospital B | | 0.792 (0.750 - 0.832) | | 0.822 (0.787 - 0.857) | | 0.871 (0.838 - 0.907) | |
| Hospital C | | 0.789 (0.747 - 0.829) | | 0.851 (0.816 - 0.881) | | 0.871 (0.835 - 0.907) | |
| Overall | | 0.806 (0.764 - 0.845) | | 0.862 (0.827 - 0.894) | | 0.874 (0.838 - 0.910) | |
| Sensitivity (Original) | | Validation Cohort | | | | | |
| Development Cohort | | Hospital A | | Hospital B | | Hospital C | |
| Hospital A | | 0.883 (0.740 - 0.948) | | 0.807 (0.674 - 0.927) | | 0.921 (0.726 - 0.945) | |
| Hospital B | | 0.909 (0.714 - 0.974) | | 0.879 (0.807 - 0.939) | | 0.849 (0.726 - 0.973) | |
| Hospital C | | 0.779 (0.675 - 0.896) | | 0.855 (0.747 - 0.927) | | 0.904 (0.821 - 0.973) | |
| Overall | | 0.844 (0.727 - 0.948) | | 0.891 (0.747 - 0.963) | | 0.877 (0.767 - 0.973) | |
| Sensitivity (SMOTE) | | Validation Cohort | | | | | |
| Development Cohort | | Hospital A | | Hospital B | | Hospital C | |
| Hospital A | | 0.772 (0.712 - 0.826) | | 0.812 (0.754 - 0887) | | 0.843 (0.741 - 0.932) | |
| Hospital B | | 0.737 (0.665 - 0.876) | | 0.855 (0.802 - 0.903) | | 0.898 (0.809 - 0.945) | |
| Hospital C | | 0.778 (0.613 - 0.892) | | 0.780 (0.700 - 0.887) | | 0.870 (0.816 - 0.918) | |
| Overall | | 0.747 (0.680 - 0.881) | | 0.802 (0.738 - 0.887) | | 0.884 (0.782 - 0.952) | |
| Specificity (Original) | | Validation Cohort | | | | | |
| Development Cohort | | Hospital A | | Hospital B | | Hospital C | |
| Hospital A | | 0.795 (0.787 - 0.909) | | 0.852 (0.692 - 0.954) | | 0.899 (0.767 - 0.943) | |
| Hospital B | | 0.720 (0.711 - 0.897) | | 0.807 (0.801 - 0.813) | | 0.878 (0.734 - 0.963) | |
| Hospital C | | 0.898 (0.798 - 0.921) | | 0.859 (0.797 - 0.928) | | 0.814 (0.907 - 0.822) | |
| Overall | | 0.812 (0.715 - 0.916) | | 0.840 (0.777 - 0.942) | | 0.909 (0.781 - 0.934) | |
| Specificity (SMOTE) | | Validation Cohort | | | | | |
| Development Cohort | | Hospital A | | Hospital B | | Hospital C | |
| Hospital A | | 0.872 (0.819 - 0.918) | | 0.918 (0.826 - 0.956) | | 0.897 (0.788 - 0.967) | |
| Hospital B | | 0.858 (0.704 - 0.919) | | 0.847 (0.798 - 0.902) | | 0.846 (0.788 - 0.923) | |
| Hospital C | | 0.808 (0.679 - 0.944) | | 0.929 (0.820 - 0.972) | | 0.865 (0.807 - 0.916) | |
| Overall | | 0.876 (0.728 - 0.925) | | 0.923 (0.837 - 0.962) | | 0.871 (0.788 - 0.948) | |
| PPV (Original) | Validation Cohort | | | | | |  |
| Development Cohort | Hospital A | | Hospital B | | Hospital C | |  |
| Hospital A | 0.019 (0.017 - 0.038) | | 0.027 (0.014 - 0.072) | | 0.051 (0.025 - 0.087) | |  |
| Hospital B | 0.014 (0.013 - 0.033) | | 0.022 (0.020 - 0.023) | | 0.044 (0.023 - 0.119) | |  |
| Hospital C | 0.031 (0.016 - 0.042) | | 0.030 (0.021 - 0.054) | | 0.030 (0.029 - 0.034) | |  |
| Overall | 0.021 (0.014 - 0.038) | | 0.025 (0.019 - 0.063) | | 0.058 (0.028 - 0.079) | |  |
| PPV (SMOTE) | Validation Cohort | | | | | |  |
| Development Cohort | Hospital A | | Hospital B | | Hospital C | |  |
| Hospital A | 0.866 (0.819 - 0.910) | | 0.908 (0.836 - 0.948) | | 0.882 (0.802 - 0.956) | |  |
| Hospital B | 0.733 (0.681 - 0.831) | | 0.852 (0.808 - 0.897) | | 0.849 (0.800 - 0.913) | |  |
| Hospital C | 0.831 (0.764 - 0.936) | | 0.917 (0.826 - 0.965) | | 0.859 (0.811 - 0.907) | |  |
| Overall | 0.876 (0.792 - 0.925) | | 0.915 (0.844 - 0.956) | | 0.864 (0.801 - 0.940) | |  |
| NPV (Original) | | Validation Cohort | | | | | |
| Development Cohort | | Hospital A | | Hospital B | | Hospital C | |
| Hospital A | | 0.999 (0.998 - 0.999) | | 0.999 (0.998 - 0.999) | | 0.998 (0.998 - 0.999) | |
| Hospital B | | 0.999 (0.998 - 0.999) | | 0.999 (0.998 - 0.999) | | 0.998 (0.998 - 0.999) | |
| Hospital C | | 0.999 (0.998 - 0.999) | | 0.999 (0.998 - 0.999) | | 0.999 (0.998 - 0.999) | |
| Overall | | 0.999 (0.998 - 0.999) | | 0.999 (0.998 - 0.999) | | 0.999 (0.998 - 0.999) | |
| NPV (SMOTE) | | Validation Cohort | | | | | |
| Development Cohort | | Hospital A | | Hospital B | | Hospital C | |
| Hospital A | | 0.781 (0.736 - 0.826) | | 0.829 (0.784 - 0.884) | | 0.857 (0.792 - 0.928) | |
| Hospital B | | 0.859 (0.775 - 0.913) | | 0.852 (0.806 - 0.897) | | 0.900 (0.830 - 0.944) | |
| Hospital C | | 0.751 ( 0.665 - 0.850) | | 0.807 (0.757 - 0.881) | | 0.877 (0.830 - 0.921) | |
| Overall | | 0.744 (0.697 - 0.836) | | 0.822 (0.777 - 0.882) | | 0.889 (0.818 - 0.945) | |

SMOTE : Synthetic minority over-sampling technique; AUROC : Area under receiver operating curve; PPV : Positive predicted value ; NPV : Negative predicted value

**Supplementary Table6. List candidate Variables and definitions.**

| **Category** | **Candidate Variables** | **Description** |
| --- | --- | --- |
| Demographic | Sex | Female and male |
|  | Age | Age in years |
| Administrative | Day of week | Classified either as Monday, Midweek (Tuesday, Wednesday, Thursday), Friday, or weekend |
|  | Shift time | 8-hour shift times including 8am to 4pm, 4pm to midnight, and midnight to 8am |
| Clinical Data | Triage Categories | Initial severity classification based on severity classification guidelines when visiting the emergency department. Classified 1(most severe) to 5(less severe). |
|  | Consciousness | The initial condition of a patient in the emergency room is measured by arousal, self-awareness, response to environmental signals, or sensory stimulation |
|  | Route of arrival | Route of visiting emergency department. |
|  | Mode of transport | main means of transportation used when visiting an emergency department |
|  | Pulse | Initially measured the number of heart beats per minute from nursing assessment. |
|  | Systolic Blood pressure, | Initially measured systolic blood pressure from nursing assessment. |
|  | Diastolic Blood Pressure | Initially measured diastolic blood pressure from nursing assessment. |
|  | Respiration | Initially measured the number of breaths taken per minute from nursing assessment. |
|  | SPo2 | Initially measured peripheral capillary oxygen  percentage saturation from nursing assessment. |
|  | Temperature | Initially measured body temperature from nursing assessment. |

**Supplementary Table7. List candidate comorbidities ICD10 and SNOMED CT concept id.**

| **Comorbidities** | **ICD 10** | **SNOMED CT concept ID** |  |
| --- | --- | --- | --- |
| Myocardial infarction | I21.x, I22.x, I25.2 | 312327,4108217,314666 |  |
| Congestive heart failure | I09.9, I11.0, I13.0, I13.2, I25.5, I42.0, I42.5–I42.9, I43.x, I50.x, P29.0 | 320746, 316139, 319825, 314378, 439696, 439694, 4110961, 4163710, 4190773, 318773, 321319, 4172864 |  |
| Peripheral vascular disease | I70.x, I71.x, I73.1, I73.8, I73.9, I77.1, I79.0, I79.2, K55.1, K55.8, K55.9, Z95.8, Z95.9 | 318443, 4187240, 312939, 321052, 317305, 317585, 44782775, 199064, 4134603 |  |
| Stroke | G45.x, G46.x, H34.0, I60.x– I69.x | 373503, 381591, 432923, 376713, 42538062, 443454, 381316, 43022059, 381591, 434056 |  |
| Dementia | F00.x–F03.x, F05.1, G30.x, G31.1 | 378419 ,443605, 374888, 4182210, 372608, 378419, 37111242, 373179 |  |
| Chronic pulmonary disease | I27.8, I27.9, J40.x–J47.x, J60.x– J67.x, J68.4, J70.1, J70.3 | 256451, 255841, 255841, 261325, 255573, 317009, 37116845, 256449, 252946, 256450, 442125, 254389, 259044, 4112676, 259044, 444084, 4307356, 3655113, 252348, 4112814 |  |
| Rheumatoid disease | M05.x, M06.x, M31.5, M32.x–M34.x, M35.1, M35.3, M36.0 | 4035611, 80809, 257628, 80182, 134442, 4343935, 4079978, 255348, 4344161 |  |
| Diabetes without complications | K25.x–K28.x | 201254, 439770, 435216, 435216, 443412, 201826, 443734, 443732, 4193704, 4221933, 4096041, 4327944, 4096042, 443727, 442793, 4008576, 443735, 442793, 442793 |  |
| Diabetes with complication | E10.0, E10.1, E10.6, E10.8, E10.9, E11.0, E11.1, E11.6, E11.8, E11.9,E12.0, E12.1, E12.6, E12.8, E12.9, E13.0, E13.1, E13.6, E13.8, E13.9, E14.0, E14.1, E14.6, E14.8, E14.9 | 200687, 42538169, 377821, 318712, 435216, 443731, 443733, 4099652, 443729, 443732, 4096670, 4224419, 4224879, 4096671, 192279, 443767, 443730, 321822, 40480000, 192279 |  |
| Hemiplegia or paraplegia | E10.2–E10.5, E10.7, E11.2– E11.5, E11.7, E12.2–E12.5, E12.7, E13.2–E13.5, E13.7, E14.2– E14.5, E14.7 | 374022, 4104204, 37019108, 192901, 132617, 134031, 372880, 195240, 379012, 381548, 4102342, 374377 |  |
| Kidney disease | G04.1, G11.4, G80.1, G80.2, G81.x, G82.x, G83.0–G83.4, G83.9 | 46271022, 192359, 443919, 439695, 252365, 4056462, 4059463, 4059584, 4055899, 4056480, 4298809, 4146996, 4284491, 433257, 36717583, 4272486, 4301680, 4032243, 42539502, 4019967 | |
| Local tumor, leukemia, and lymphoma | I12.0, I13.1, N03.2–N03.7, N05.2–N05.7, N18.x, N19.x, N25.0, Z49.0–Z49.2, Z94.0, Z99.2 | 4155171, 256633, 437498, 140950, 135750, 134290, 25189, 434588, 4247836, 31509, 442131, 259748, 26052, 4177112, 4311499, 438368, 438693, 4247331, 76914, 4180312, 76914, 141232, 4033891, 434584, 376918, 376918, 376647, 4162253, 195197, 196048, 196359, 4162860, 45770892, 200051, 201238, 36715801, 195483, 200962, 433716, 197507, 198985, 195480, 200054, 196360, 76349, 442131, 4002340, 380055, 4247822, 133424, 198104, 133420, 4114221, 4038835, 4147411, 4003830, 4212994, 4038838, 437233, 132853, 140666, 321526, 317510, 4147164, 4094409 | |
| Metastatic solid tumor | C00.x–C26.x, C30.x–C34.x, C37.x–C41.x, C43.x, C45.x–C58.x, C60.x–C76.x, C81.x–C85.x, C88.x, C90.x–C97.x | 318096, 4147162, 35225597, 36769180, 432851, 443392 | |
| Mild liver disease | C77.x–C80.x | 4012113, 200763, 4267417, 193256, 4340383, 4340385, 196463, 201612, 4059298, 4055225, 4059299, 4058695, 4059290, 4058696, 194417, 4240725, 194984, 42537742 | |
| Severe liver disease | B18.x, K70.0–K70.3, K70.9, K71.3–K71.5, K71.7, K73.x, K74.x, K76.0, K76.2–K76.4, K76.8, K76.9, Z94.4 | 28779, 22340, 4237824, 22340, 4340386, 4026136, 4340390, 4245975, 4277276, 192680, 196455 | |
| Myocardial infarction | I85.0, I85.9, I86.4, I98.2, K70.4, K71.1, K72.1, K72.9, K76.5, K76.6, K76.7 | 312327,4108217,314666 | |

ICD : International classification of Disease ; SNOMED CT: Systematized nomenclature of medicine–clinical terms

**Supplementary Table8.** TRIPOD Checklist: Prediction Model Development and Validation

| **Section/Topic** | **Item** |  | **Checklist Item** | **Page** |
| --- | --- | --- | --- | --- |
| **Title and abstract** | | | | |
| Title | 1 | D;V | Identify the study as developing and/or validating a multivariable prediction model, the target population, and the outcome to be predicted. | 1 |
| Abstract | 2 | D;V | Provide a summary of objectives, study design, setting, participants, sample size, predictors, outcome, statistical analysis, results, and conclusions. | 2 |
| Introduction | | | | |
| Background and objectives | 3a | D;V | Explain the medical context (including whether diagnostic or prognostic) and rationale for developing or validating the multivariable prediction model, including references to existing models. | 3 |
|  | 3b | D;V | Specify the objectives, including whether the study describes the development or validation of the model or both. | 3 |
| Methods | | | | |
| Source of data | 4a | D;V | Describe the study design or source of data (e.g., randomized trial, cohort, or registry data), separately for the development and validation data sets, if applicable. | 11 |
|  | 4b | D;V | Specify the key study dates, including start of accrual; end of accrual; and, if applicable, end of follow-up. | 11 |
| Participants | 5a | D;V | Specify key elements of the study setting (e.g., primary care, secondary care, general population) including number and location of centres. | 11 |
|  | 5b | D;V | Describe eligibility criteria for participants. | 11 |
|  | 5c | D;V | Give details of treatments received, if relevant. | Not applicated |
| Outcome | 6a | D;V | Clearly define the outcome that is predicted by the prediction model, including how and when assessed. | 11 |
|  | 6b | D;V | Report any actions to blind assessment of the outcome to be predicted. | Not applicated |
| Predictors | 7a | D;V | Clearly define all predictors used in developing or validating the multivariable prediction model, including how and when they were measured. | 11 |
|  | 7b | D;V | Report any actions to blind assessment of predictors for the outcome and other predictors. | Not applicated |
| Sample size | 8 | D;V | Explain how the study size was arrived at. | 11 |
| Missing data | 9 | D;V | Describe how missing data were handled (e.g., complete-case analysis, single imputation, multiple imputation) with details of any imputation method. | Not applicated |
| Statistical analysis methods | 10a | D | Describe how predictors were handled in the analyses. | 11-13 |
|  | 10b | D | Specify type of model, all model-building procedures (including any predictor selection), and method for internal validation. | 11-13 |
|  | 10c | V | For validation, describe how the predictions were calculated. | 11-13 |
|  | 10d | D;V | Specify all measures used to assess model performance and, if relevant, to compare multiple models. | 11-13 |
|  | 10e | V | Describe any model updating (e.g., recalibration) arising from the validation, if done. | 11-13 |
| Risk groups | 11 | D;V | Provide details on how risk groups were created, if done. | Not applicated |
| Development vs. validation | 12 | V | For validation, identify any differences from the development data in setting, eligibility criteria, outcome, and predictors. | 11-13 |
| Results | | | | |
| Participants | 13a | D;V | Describe the flow of participants through the study, including the number of participants with and without the outcome and, if applicable, a summary of the follow-up time. A diagram may be helpful. | 3-4 |
|  | 13b | D;V | Describe the characteristics of the participants (basic demographics, clinical features, available predictors), including the number of participants with missing data for predictors and outcome. | 3-7 |
|  | 13c | V | For validation, show a comparison with the development data of the distribution of important variables (demographics, predictors and outcome). | 3-7 |
| Model development | 14a | D | Specify the number of participants and outcome events in each analysis. | 11-13 |
|  | 14b | D | If done, report the unadjusted association between each candidate predictor and outcome. | 11-13 |
| Model specification | 15a | D | Present the full prediction model to allow predictions for individuals (i.e., all regression coefficients, and model intercept or baseline survival at a given time point). | 11-13 |
|  | 15b | D | Explain how to the use the prediction model. | 11-13 |
| Model performance | 16 | D;V | Report performance measures (with CIs) for the prediction model. | 9-10 |
| Model-updating | 17 | V | If done, report the results from any model updating (i.e., model specification, model performance). | 9-10 |
| Discussion | | | | |
| Limitations | 18 | D;V | Discuss any limitations of the study (such as nonrepresentative sample, few events per predictor, missing data). | 10 |
| Interpretation | 19a | V | For validation, discuss the results with reference to performance in the development data, and any other validation data. | 10 |
|  | 19b | D;V | Give an overall interpretation of the results, considering objectives, limitations, results from similar studies, and other relevant evidence. | 10 |
| Implications | 20 | D;V | Discuss the potential clinical use of the model and implications for future research. | 10 |
| Other information | | | | |
| Supplementary information | 21 | D;V | Provide information about the availability of supplementary resources, such as study protocol, Web calculator, and data sets. | 17-32 |
| Funding | 22 | D;V | Give the source of funding and the role of the funders for the present study. | 16 |

*Items relevant only to the development of a prediction model are denoted by D, items relating solely to a validation of a prediction model are denoted by V, and items relating to both are denoted D;V. We recommend using the TRIPOD Checklist in conjunction with the TRIPOD Explanation and Elaboration document.
